# An Improved Weighted and Location-Based Clustering Scheme for Flying Ad Hoc Networks

**DOI:** 10.3390/s22093236

**Published:** 2022-04-22

**Authors:** Xinwei Yang, Tianqi Yu, Zhongyue Chen, Jianfeng Yang, Jianling Hu, Yingrui Wu

**Affiliations:** 1 School of Electronic and Information Engineering, Soochow University, Suzhou 215006, China; 20204228021@stu.suda.edu.cn (X.Y.); tqyu@suda.edu.cn (T.Y.); chenzy@suda.edu.cn (Z.C.); jlhu@suda.edu.cn (J.H.); 1928401099@stu.suda.edu.cn (Y.W.); 2 School of Electronic and Information Engineering, Wuxi University, Wuxi 214105, China

**Keywords:** unmanned aerial vehicle (UAV), K-means++ clustering, cluster head selection, cluster maintenance, flying ad hoc network (FANET)

## Abstract

Flying ad hoc networks (FANETs) have been gradually deployed in diverse application scenarios, ranging from civilian to military. However, the high-speed mobility of unmanned aerial vehicles (UAVs) and dynamically changing topology has led to critical challenges for the stability of communications in FANETs. To overcome the technical challenges, an Improved Weighted and Location-based Clustering (IWLC) scheme is proposed for FANET performance enhancement, under the constraints of network resources. Specifically, a location-based K-means++ clustering algorithm is first developed to set up the initial UAV clusters. Subsequently, a weighted summation-based cluster head selection algorithm is proposed. In the algorithm, the remaining energy ratio, adaptive node degree, relative mobility, and average distance are adopted as the selection criteria, considering the influence of different physical factors. Moreover, an efficient cluster maintenance algorithm is proposed to keep updating the UAV clusters. The simulation results indicate that the proposed IWLC scheme significantly enhances the performance of the packet delivery ratio, network lifetime, cluster head changing ratio, and energy consumption, compared to the benchmark clustering methods in the literature.

## 1. Introduction

Unmanned aerial vehicles (UAVs) have been pervasively used in civilian and military fields, such as collaborative formation, mission reconnaissance, precision agriculture, material distribution, and environmental monitoring [1]. However, the computational and communication capabilities of a single UAV cannot meet the increasing requirements of such applications [2,3]. Additionally, because of the rapid development of wireless communication technology, the miniaturization, intelligence, and networking of UAVs have become a research trend [4].

Under such a situation, flying ad hoc networks (FANETs), a new research field of ad hoc networks, have developed as a promising networking paradigm. FANETs share properties with mobile ad hoc networks (MANETs) and their sub-classes, such as vehicular ad hoc networks (VANETs) and wireless sensor networks (WSNs). However, FANETs have the features of high mobility, scalability, three-dimensional (3D) deployment, and frequent topology changes. Furthermore, UAVs as the network nodes are capable of transmitting information, exchanging data packets, and automatically establishing a wireless network in the air. The velocity and density of UAVs are greater than other ad hoc networks. These features can lead to the instability of UAV swarms, which makes it difficult to design a stable and effective scheme for FANETs [5].

The network communications can be affected by several issues, including unstable link connections between UAVs, limited communication range between the ground control station (GCS) and UAVs, and constrained resource supply [6]. UAVs are equipped with polymer lithium batteries that sustain the flight time. Moreover, the high mobility of UAVs exacerbates frequent topology changes in the FANET. Thus, the effective deployment and management of UAVs can significantly enhance network stability.

Clustering, as a method of network organization, can improve the network performance by increasing the network lifetime and packet delivery ratio (PDR), while reducing the routing overhead. UAV swarms can accomplish efficient deployment and stable communication in the clustering structure. Specifically, the overall network is separated into clusters in the clustering process. A cluster is composed of a cluster head (CH) and cluster members (CMs). The burden of CHs is heavy in clustering networks because they must manage the changes in CMs and transmit packets. The CHs are chosen by a selection algorithm that takes into account the significant physical variables of the candidate network nodes. Because the initial cluster formation and CH selection are critical to the clustering structure, it is necessary to develop an effective clustering scheme for enhancing the Quality of Service (QoS) of FANETs.

To address the related issues, an Improved Weighted and Location-based Clustering (IWLC) scheme is proposed to ensure effective clustering, link stability and network lifetime in this paper. A location-based K-means++ clustering algorithm is first developed to determine the number of clusters in a FANET and set up the initial clusters. Secondly, we propose a CH selection algorithm, where the remaining energy ratio, adaptive node degree, relative mobility, and average distance of a UAV node are all involved in the selection criteria. Furthermore, a cluster maintenance strategy is presented for enhancing the stability and robustness of the clustering network. Simulations have been conducted to analyze the performance of the proposed IWLC scheme. The simulation results indicate that the proposed scheme outperforms the benchmark clustering schemes in the literature, regarding the network lifetime, PDR, cluster head changing ratio, throughput and energy consumption. The main contributions of this paper are summarized as follows:

(1) An improved weighted and location-based clustering (IWLC) scheme has been proposed for FANETs, to improve the network performance. The proposed IWLC scheme is composed of an initial cluster formation, CH selection, and cluster maintenance.
(2) We have proposed a weighted summation-based CH selection algorithm, where the remaining energy ratio, adaptive node degree, relative mobility, and average distance of a UAV node are all involved in the selection criteria. The UAV node with the maximum weight will be selected as the CH.
(3) The proposed IWLC scheme has been comprehensively evaluated by simulation experiments, from the perspectives of the influence of weight settings and the comparison with the benchmark clustering schemes.

The remainder of the paper is structured in the following manner. Section 2 summarizes the related work. The system model is introduced in Section 3. In Section 4, the proposed IWLC scheme is presented in detail. In Section 5, the performance of the IWLC scheme is evaluated by simulations and compared to the benchmark clustering schemes. Finally, the contributions of the work are summarized in Section 6.

## 2. Related Works

Several research efforts have been dedicated to the clustering schemes for FANETs. This section provides an overview of the existing literature and an in-depth analysis of their principles and remaining technical limitations.

The lowest ID clustering algorithm (LID) was proposed by Gerla et al. [7], where UAV nodes with lower IDs were selected as CHs. This algorithm is widely used, owing to its low time complexity and overhead of maintaining the clustering structure. However, the UAV nodes with lower IDs run out of battery faster than other nodes, because they act as the CHs and consume more energy, which finally leads to faster network partitions. LEACH-C (Low Energy Adaptive Clustering Hierarchy-Centralized) [8] is a classic centralized CH generation algorithm, where the base station is responsible for selecting CHs in the algorithm. The hybrid energy-efficient distributed clustering algorithm (HEED) [9] states that extending network lifetime and load balance are the main requirements in FANETs. The network lifetime is extended by evenly distributing energy consumption across the network. The CH generation is carried out within finite iterations in HEED, which minimizes the control message overhead. To enhance the QoS performance in FANETs, an SDN-MQTT (Software Defined Networking-Message Queue Telemetry Transport) [10] scheme is proposed. The paper presents a hybrid structure of SDN and MQTT for battlefield UAV clusters, which is scalable and controllable for network topology and payloads. The SDN-MQTT structure can support flexible packet transmission and improve communication security.

In [11], the authors proposed a predictive scheme combined with unicasting and geocasting routes using trajectory information, which keeps track of the changing topology and increases link stability. In [12], the authors used the relative typical node degree to evaluate the stability of nodes when selecting CHs, but did not take the average distance between nodes into account. The author in [13] proposed an Energy Aware Link-based Clustering (EALC) route to improve network performance. EALC uses an improved K-means fitness algorithm, which adopts energy level and average distance for optimal CH selection. EALC achieves routing calculation optimization and energy efficiency, but it only considers UAVs with low mobility. A mobility prediction-based clustering algorithm (MPCA) estimated the link lifetime based on the location and speed information of UAV nodes [14]. The algorithm combines average connectivity, link expiation time, and the probability that a candidate node maintains its current status in CH selection, which can prolong the network lifetime and PDR, but the routing overhead of the algorithm is much higher because of the frequent updating of the network topology. In [15], a mobility control-based clustering scheme (MOOC) was proposed to maintain the clustering structure in FANETs. The weighted clustering algorithm (WCA) considered the influence of different physical factors [16,17,18]. This algorithm evaluates the ability of nodes to run for the CH and can adjust the corresponding physical factors, based on the different network characteristics. The algorithm in [19] selects only one CH in each cycle, increasing network routing overhead and time complexity. Wang [20] solved the WCA’s issue of lacking network communication security in CH selection, and improved the encrypted authentication mechanism in FANETs. The authors in [21] proposed a weighted stable clustering algorithm (WSCA), which comprehensively considered four physical factors, and each node in the network maintained its own neighbor table for CH election and link communication. Shi et al. presented a cluster-based location-aided routing protocol (CBLADSR) to address network security and stability issues [22], which is a heuristic-based clustering scheme to select CHs and achieve cluster formation. The main task of CBLADSR is to reduce end-to-end delay in the FANET.

Some researchers have been inspired by biological behaviors and have applied these to clustering the FANETs. In [23], an ant colony optimization (ACO) clustering scheme was proposed for optimal clustering and data transmission in FANETs. CHs are vertices in the search space, and each round provides the set of CHs from specific environments. The algorithm uses two objective functions to evaluate the fitness value of each cycle, namely the Euclidean distance and the delta difference. In [24], the grey wolf optimization (GWO) algorithm was proposed for an energy-efficient routing protocol. In [25,26], a particle swarm optimization (PSO) algorithm was proposed to perform a clustering strategy, which simulated swarm intelligence to solve optimization problems. In [27], a bio-inspired clustering (BIC) algorithm was proposed for FANETs. The algorithm is a hybrid scheme of glowworm swarm optimization (GSO) and the krill herd (KH) algorithm to achieve efficient clustering management. The UAV node with the highest fitness value is selected as the CH in a cluster, while the remainders are CMs, which can improve adaptability and reliability in FANETs.

Table 1 summarizes the performance positioning of the discussed clustering schemes in FANETs. From Table 1, we can observe that the proposed IWLC scheme comprehensively considers the stability, mobility, energy efficiency, clustering overhead, and effective data transmission in the clustering network.

## 3. Preliminaries

In this section, the overall system model and the structure of the Hello message are provided. Furthermore, the notations used in this paper are listed in Table 2.

### 3.1. System Model

**Figure 1:**
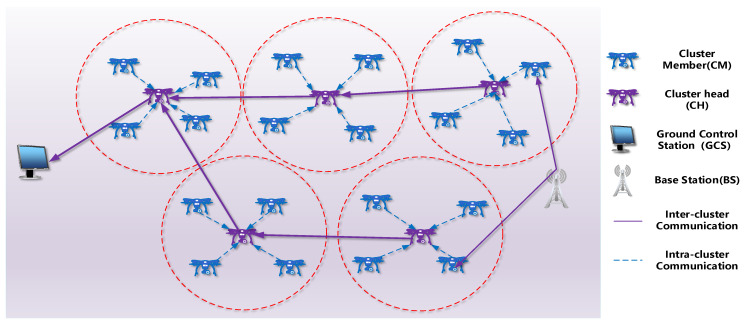
UAV clustering network structure.

The hierarchical system architecture is depicted in Figure 1. In the ground level, GCSs are deployed. We assume that the GCS has knowledge of the topology of FANETs [28]. The GCS formulates clusters and maintains the backbone routing table for communicating with CHs. GCS broadcasts central control commands to all UAVs through CHs.

The aerial level is dominated by UAVs. In the system, it is assumed that all the UAVs are equipped with location-aware components and wireless communication interfaces. Specifically, UAVs are embedded with inertial measurement units (IMUs) and GPS chipsets for measuring velocity and location information and have the capability of routing. The UAVs are then clustered according to the proposed clustering scheme. Each cluster is composed of a CH and CMs. The CHs are responsible for inter- and intra-cluster communications, cluster management, and communications with GCSs. The CH maintains its cluster by updating the list of CMs, according to the HELLO message received from the CMs. The CMs perform missions under the command control of CHs. Furthermore, the CHs are adaptively updated, and the CMs are candidates for CH selection.

### 3.2. HELLO Message

**Figure 2:**
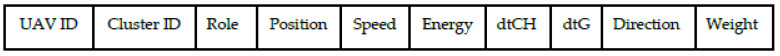
HELLO message structure.

In the system, UAV nodes exchange information by sending HELLO messages. The structure of the HELLO message is presented in Figure 2. The HELLO message contains the following fields:

- UAV ID ID and Cluster ID CID refer to the identification of the UAV and cluster.
- Role distinguishes a UAV as a CH or a CM in a cluster. It is a binary field, where 1 refers to CH and 0 is for CM.
- Position, Speed and Energy of a UAV is represented by (x,y,z), v and E.
- *dtCH* and dtG means the distance between the UAV and its CH and the GCS, respectively.
- Direction θ and φ is the flying direction of the UAV.
- Weight W details the weight of a CH candidate in the CH selection phase.

## 4. An Improved Weighted and Location-Based Clustering Scheme

### 4.1. Cluster Formation

The initial number of clusters determination is as follows: In the clustering process, to reduce the clustering overhead and utilize the bandwidth efficiently, the primary number of clusters needs to be optimally determined for the initial clustering center selection. According to [29], the throughput of each CM is

(1) $$T_{CM}=\Theta (B_{1}/\sqrt{N/k})$$

In addition, the throughput of each CH is

(2) $$T_{CH}=\Theta (B_{2}/\sqrt{k})$$

where N is the number of UAVs, and k is the number of clusters. Θ() is the asymptotically tight bound. $B_{1}$ and $B_{2}\;$are the bandwidth of intra- and inter-cluster communications, respectively. It assumes that the network traffic is uniformly distributed. Because of the throughput balance between the inter-cluster and the intra-cluster communications, the proportion of $T_{CM}\;$of a CH used for traffic in/out to other clusters is (k−1)/k, and the portion should be smaller or equal to $T_{CH}$ of that CH [30],

(3) $$\frac{k-1}{k}T_{CM}\leq T_{CH}$$

When $\frac{k-1}{k}T_{CH}$ reaches the maximum throughput, namely, when inequality Equation (3) holds, the primary number of clusters $k^{*}$ is obtained as

(4) $$k^{*}=\frac{B_{2}}{B_{1}}\sqrt{N}+1$$

If N is large enough, the number of clusters is approximated to

(5) $$k^{*}=\frac{B_{2}}{B_{1}}\sqrt{N}$$

Based on the primary value $k^{*}$, K-means++ clustering algorithm is further developed to divide N UAVs into clusters. The K-means++ clustering algorithm can reduce the error of the clustering result and the computational complexity. The UAV nodes are modeled as a point set $S=\left\{s_{1},s_{2},\ldots s_{i}\ldots ,s_{N}\right\}.$ The procedures of the algorithm are stated as follows.

*The initial clustering center selection is as follows:* An initial clustering center $c_{1}$ is randomly selected from set S, following the uniform distribution. The minimum distance, namely the minimum value of the Euclidean distances between a point $s_{i}$ and the currently selected clustering centers is denoted as $D\left(s_{i}\right)$. Then, we calculate the probability that a point $s_{i}$ in S is selected as the next clustering center by using the following equation:

(6) $$P(s_{i})=\frac{D{\left(s_{i}\right)}^{2}}{\sum_{s_{i}\in S}D{\left(s_{i}\right)}^{2}}$$

We select the point with the highest probability as the next center$\;c_{i}.$ We repeat the above process until a total $k^{*}$ centers have been chosen, which can be expressed as the set $C=\left\{c_{1},c_{2},\ldots ,c_{k^{*}}\right\}$. The $k^{*}$ centers will be used as the original clustering centers for the K-means++ clustering.

*The cluster formation is as follows:* With the original clustering centers, the set S can be divided into $k^{*}$ clusters by finding the closest clustering center. The minimum distance from a point $s_{i}$ in S to a clustering center can be expressed as $d_{cs_{i}}$.

(7) $$d_{cs_{i}}=\min(d_{c_{1}s_{i}},d_{c_{2}s_{i}},\ldots ,d_{c_{k^{*}}s_{i}})$$

The point $s_{i}$ should be clustered into the $n^{th}$ cluster, if $d_{cs_{i}}=d_{c_{n}s_{i}}$. Then, the clustering centers are updated using the following equation:

(8) $$C=\{\frac{1}{N_{n}}(\sum_{i=1}^{N_{n}}x_{i},\sum_{i=1}^{N_{n}}y_{i},\sum_{i=1}^{N_{n}}z_{i})\}$$

where C denotes the set of clustering centers and $N_{n}$ is the number of points in the $n^{th}\;$cluster.

The steps (7) and (8) will be repeated until C convergence. During this process, the actual number of clusters K could be less than the initial number of clusters $k^{*}$. The reason is that the adaptive clustering procedure merges the clusters close to each other, to mitigate the cluster with isolated or few numbers of nodes.

The pseudocode of the K-means++ clustering algorithm is listed in Algorithm 1.
**Algorithm 1:** K-means++ Clustering Algorithm**Input:** initial cluster number $k^{*}$, point set S
**Output:** cluster set$\;CL=\left\{CL_{1},CL_{2},\ldots CL_{i}\ldots ,CL_{K}\right\}$
/*Initialization*/
1: randomly select an initial center $c_{1}\;$uniformly from *S*
2: **Repeat**
3: choose the next clustering center $c_{i}$ by Equation (6)
4: **until** $k^{*}$ clustering centers selected
/*Cluster Formation*/
5: **Repeat**
6: cluster the points in S by Equation (7)
7: update the clustering centers by Equation (8)
8: **until**  C converges

### 4.2. Cluster Head Selection

Due to the high mobility and frequent topology changes, there are many factors that can affect the performance of FANETs, including network load, communication bandwidth, and node mobility. Therefore, when selecting a cluster head, multiple factors need to be considered comprehensively, so that the clustering scheme can adapt to complex environments.

In this paper, a cluster head selection algorithm is developed, where the relative mobility, adaptive node degree, node remaining energy, and average distance between nodes are jointly considered. A minimum threshold is set for each of the factors. When the four factors are all greater than the minimum threshold, the node is eligible to participate in the selection of CHs. The weight of the CH candidate node i is calculated as

(9) $$W_{i}=\omega_{1}E_{i}+\omega_{2}N_{i}+\omega_{3}M_{i}+\omega_{4}\frac{1}{D_{i}}$$

where $\omega_{1},\;\omega_{2},\;\omega_{3},\;\omega_{4}$ are the weights of the four factors, respectively, and $\sum_{i=1}^{4}\omega_{i}=1\;$. The specific values are determined by the requirements of the practical application scenarios. In the above weight expression, E is the remaining energy ratio, N is the adaptive node degree, M is the relative mobility, and D is the average distance between nodes. The four factors are normalized to mitigate the effect of different scaling units.

#### 4.2.1. Remaining Energy Ratio

In FANETs, the remaining energy is one of the critical factors affecting the network life. The energy of the UAV nodes is related to the number of neighbors, flight speed, and moving distance. The CH selection is regarded as a significant task for clustering because CHs are responsible for both inter- and intra-cluster communications and the management of clusters. If a node serves as the CH for a long time, its energy depletes dramatically, which reduces the lifetime of the whole network. Thus, the remaining energy needs to be involved in the CH selection criteria. In this work, the remaining energy ratio of node i is defined as

(10) $$E_{i}=\frac{E_{0}-E_{T}}{E_{0}}$$

where $E_{i}$ is the remaining energy ratio of i, $E_{0}$ is the initial energy of i and $E_{T}$ is the total energy consumption of i, which can be expressed as

(11) $$E_{T}=E_{Tx}+E_{Rx}+E_{f}$$

where$\;E_{Tx}\;$and$\;E_{Rx}\;$are the energy spent for transmitting and receiving communication, which occupies the largest proportion of total energy consumption. $E_{f}$ represents the energy consumed by flying. The communication energy consumption for transmitting m-bit packet is calculated as

(12) $$E_{T_{x}}=\left\{ \begin{array}{cc} mE_{elec}+m\varepsilon_{fs}d^{2}\; & d\;<\;d_{0}\\ mE_{elec}+m\varepsilon_{mp}d^{4}\; & d\;\geq \;d_{0} \end{array}\right.$$

where $E_{elec}$ is the energy consumption of the transceiver circuit for processing a unit bit data, $d_{0}$ is a threshold of the transmission distance, and d represents the distance between the transmitter and receiver. $\varepsilon_{fs}$ and $\varepsilon_{mp}$ are the power amplifier coefficients. The communication energy consumption for receiving m-bit packet is calculated as

(13) $$E_{Rx}=mE_{elec}$$

**Figure 3:**
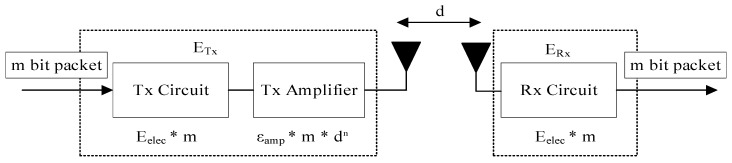
Radio energy dissipation model.

The radio energy dissipation model is shown in Figure 3. When the transmission distance is less than the threshold $d_{0}$, the free space channel model is used and the power amplifier coefficient $\varepsilon_{amp}$ is set to $\varepsilon_{fs}$. When the transmission distance is greater than or equal to the threshold $d_{0}$, the multi-diameter decay model is used and the $\varepsilon_{amp}\;$is set to $\varepsilon_{mp}$.

The flying energy consumption of node i is calculated as follows:

(14) $$E_{f}=\int_{0}^{t_{f}}P_{f}dt$$

(15) $$P_{f}=\sqrt{\frac{{\left(m_{UAVs}g\right)}^{3}}{2\pi n_{w}r_{w}\rho_{air}}}$$

where $P_{f}$ and $t_{f}$ are the UAV flying power and time, $m_{UAVs}$ is the total mass of UAVs, $\rho_{air\;}$and g are the air density and earth gravity, respectively. $n_{w}$ is the number of wings, and $r_{w}$ is the radius of wings.

#### 4.2.2. Adaptive Node Degree

Due to the limited network bandwidth resources, the number of neighbor nodes needs to be controlled within a certain range. The clustering scale is constrained by a certain condition in the CH selection. If the CH coverage range has a small number of nodes, the bandwidth cannot be fully utilized. If the CH coverage range contains a significant number of nodes, network congestion and transmission delays grow. The adaptive node degree is used in this work to avoid network congestion, which is positively correlated to the probability of being selected as CH. In [29], it states that if the distance between two nodes is less than the maximum transmission range R, they can be regarded as two R-neighbors. Furthermore, the distance can be calculated by using the position enclosed in the broadcasted HELLO message. Hence, the total number of R-neighbors of i is defined as its node degree ($Nd_{i}$), demonstrated in the following equation:

(16) $$Nd_{i}=\left|\varphi_{i}\right|=\sum_{j\in S,j\neq i}\left\{d_{ij}<R\right\}$$

where $\varphi_{i}$ is the set of neighbor nodes of i, and$\;d_{ij}$ is the average distance between node i and j. The adaptive node degree difference is defined as

(17) $$n_{i}=\left|Nd_{i}-\frac{N}{k^{*}}\right|$$

where $k^{*}$ is the optimal number of clusters determined by Equation (5). The adaptive node degree is normalized [20] by

(18) $$N_{i}=e^{-n_{i}}$$

#### 4.2.3. Relative Mobility

**Figure 4:**
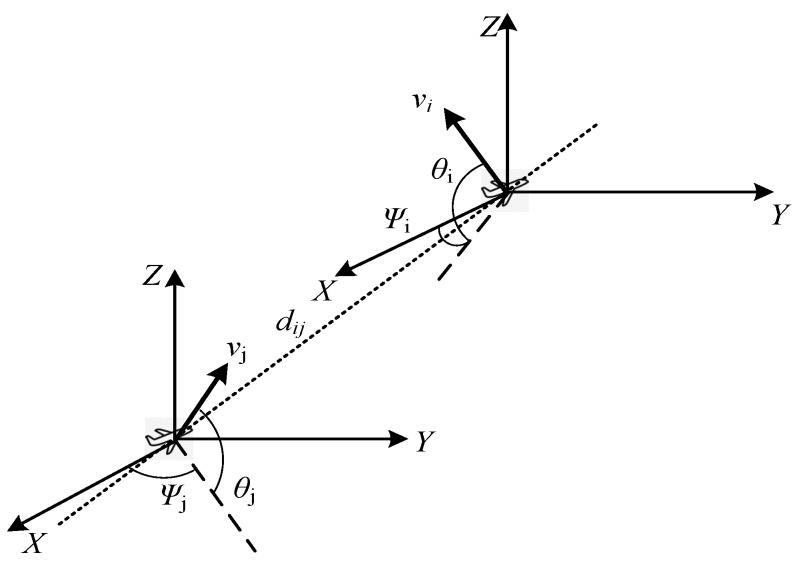
Relative geometric parameters of UAVs.

In FANETs, the relative mobility of nodes in a cluster should be controlled within a certain range to guarantee the stability and robustness of the network. The nodes with fast moving speed are not eligible to be candidates for CH selection. As mentioned above, the UAVs are equipped with IMU and GPS. In this work, a method is proposed to calculate the relative mobility of UAVs in a 3D coordinate system, by using the location and speed information. The movement state of node i and j is shown in Figure 4, and the speed differences between i and j in x, y and z axes are given as the following:

(19) $$\Delta v_{x}=v_{i}\cos\theta_{i}\cos\psi_{i}-v_{j}\cos\theta_{j}\cos\psi_{j}$$

(20) $$\Delta v_{y}=v_{i}\cos\theta_{i}\sin\psi_{i}-v_{j}\cos\theta_{j}\sin\psi_{j}$$

(21) $$\Delta v_{z}=v_{i}\sin\theta_{i}-v_{j}\sin\theta_{j}$$

The average speed difference between i and its neighbor nodes can be calculated using the following equations:

(22) $$\bar{v_{ix}}=\frac{\sum_{j=1}^{Nd_{i}}(v_{i}\cos\theta_{i}\cos\psi_{i}-v_{j}\cos\theta_{j}\cos\psi_{j})}{Nd_{i}}$$

(23) $$\bar{v_{iy}}=\frac{\sum_{j=1}^{Nd_{i}}(v_{i}\cos\theta_{i}\sin\psi_{i}-v_{j}\cos\theta_{j}\sin\psi_{j})}{Nd_{i}}$$

(24) $$\bar{v_{iz}}=\frac{\sum_{j=1}^{Nd_{i}}(v_{i}\sin\theta_{i}-v_{j}\sin\theta_{j})}{Nd_{i}}$$

(25) $$\bar{v_{i}}=\sqrt{{\bar{v_{ix}}}^{2}+{\bar{v_{iy}}}^{2}+{\bar{v_{iz}}}^{2}}$$

The variances of speed difference between i and its neighbor nodes along the XYZ axes are calculated as

(26) $${\sigma_{ix}}^{2}=\frac{\sum_{j=1}^{Nd_{i}}(\bar{v_{ix}}-\Delta v_{x}{)}^{2}}{Nd_{i}},{\sigma_{iy}}^{2}=\frac{\sum_{j=1}^{Nd_{i}}(\bar{v_{iy}}-\Delta v_{y}{)}^{2}}{Nd_{i}},{\sigma_{iz}}^{2}=\frac{\sum_{j=1}^{Nd_{i}}(\bar{v_{iz}}-\Delta v_{z}{)}^{2}}{Nd_{i}}$$

The average variance of speed difference between node i and its neighbor nodes is

(27) $${\bar{\sigma_{i}}}^{2}=\frac{\sigma_{ix}^{2}+\sigma_{iy}^{2}+\sigma_{iz}^{2}}{3}$$

Finally, the relative mobility is normalized [20] by

(28) $$M_{i}=e^{-({\bar{\sigma_{i}}}^{2}+{\bar{v_{i}}}^{2})}$$

#### 4.2.4. Average Distance

To calculate the average distance, the UAV nodes attach the GPS location information to the HELLO message and send it to neighbor nodes. The average distance between i and its neighbors can be calculated as

(29) $$d_{i}=\frac{1}{Nd_{i}}\sum_{j=1}^{Nd_{i}}\sqrt{{\left(x_{j}-x_{i}\right)}^{2}+{\left(y_{j}-y_{i}\right)}^{2}+{\left(z_{j}-z_{i}\right)}^{2}}$$

where $\left(x_{i},\;y_{i},\;z_{i}\right)$ is the 3D coordinate of the i, and $\left(x_{j},\;y_{j},\;z_{j}\right)$ is the position of its neighbor node j. The average distance is normalized [5] by

(30) $$D_{i}=\log(\frac{d_{i}}{R}+1)$$

Based on the definition of the four factors, the cluster head selection is proposed, of which the pseudocode is listed in Algorithm 2.
**Algorithm 2:** Cluster Head Selection Algorithm**Input:** cluster numbers K, clusters set $CL=\left\{CL_{1},CL_{2},\ldots CL_{i}\ldots ,CL_{K}\right\}$.
**Output:** cluster head $CH_{i}$, i=1,2,…,K
/*Initialization*/
1: Each UAV node broadcasts a HELLO message to its neighboring set φ.
/*Computation*/
2: **for** i=1: K
3: **for** each UAV node jϵ $CL_{i}$
4: calculate the weight $W_{j}$ by using Equation (9)
5: **end for**
6: $CLbest=\underset{j}{argmax}\{W_{j}\}$
7: $CH_{i}$ = CLbest
8: broadcast $CH_{i}$ claim
9: **end for**

### 4.3. Cluster Maintenance

After the initial formation of clusters, dynamic topology changes frequently occur in FANETs. Thus, it is significant to enhance the robustness of the clustering network in dynamic environments. In this work, cluster maintenance is proposed to keep the stability of the cluster structure and improve the network performance. The following analyzes the cluster maintenance in different situations.

To maintain the clusters effectively, the CHs broadcast their HELLO messages to their CMs periodically with a period T, and a CM replies with an ACK (acknowledgement) message to its CH, immediately after receiving the HELLO message. If the CH cannot receive an ACK from the CM for β periods, the CM is considered to not be in the transmission range and is depleted from the CM list. If a CH leaves the cluster, a CH re-selection process is triggered.

If a new node (including a newly added node and the node that has left the original cluster) wishes to join the clustering network, it sends its HELLO message to the nearest CH. The CH checks whether the remaining energy of the new node is greater than the threshold $E_{th}$, which is set to 20% of the initial energy in the paper. If it meets the requirements, the CH replies with an ACK to the new node. The pseudocode of cluster maintenance is listed in Algorithm 3.
**Algorithm 3:** Cluster Maintenance1: **if** ACK from $CM_{j}$ is not heard by CH
2:  deplete $CM_{j}$ from CM list
3: **end if**
4: **if**
$CH_{i}$ leaves the cluster
5:  execute Algorithm 2
6: **end if**
7: **if** a new node requests to join the cluster
8:  the new node sends HELLO message to $CH_{i}$
9:  **if** (remaining energy of the new node > $E_{th}$)
10:   $CH_{i}\;$reply ACK and agree to join
11:  **else**
12:   ignore the request
13:  **end if**
14: **end if**

## 5. Performance Evaluation

In this section, we present the performance evaluation of the proposed IWLC scheme. First, the simulation settings are presented in detail and IWLC is analyzed by different weight settings. Then, the performance of the proposed IWLC is evaluated and compared with the benchmark methods, namely, WSCA and ACO. The metrics used in the simulation are PDR, number of clusters, network lifetime, cluster head changing ratio, and energy consumption.

### 5.1. Simulation Environment

The simulation parameters are presented in Table 3. The simulation scenario is a 3D space of 2000 m × 2000 m × 1000 m. The transmission range of UAVs is set to 300 m. It means that the receiver nodes can decode the received signals correctly, when the distance between the sender and the receiver nodes is less than 300 m. The number of UAV nodes varies from 20 to 100, and the speed of UAV nodes ranges from 10 to 30 m/s. We employ the IEEE 802.11n radio standard [31], operating at the 2.4 GHz and the 5 GHz frequency band for intra-cluster and inter-cluster communications. In the radio energy dissipation model, the energy parameters of communication consumption are set as the following: $E_{elec}=$ 50 nJ/bit, $\varepsilon_{fs}=100\;\mathrm{pJ}/\mathrm{bit}/m^{2}$, and $\;\varepsilon_{mp}=0.01\;\mathrm{pJ}/\mathrm{bit}/m^{4}.\;$The energy parameters of flying consumption are set as the following:$\;m_{UAVs}=0.7\;\mathrm{kg},\;n_{w}=4,\;$and $r_{w}=0.15\;m.\;$The simulations are run on a Windows 10 operating system, with a 2.30 GHz Intel Core i7-10875H CPU and 3.2GHz DDR4 RAM.

### 5.2. Analysis of IWLC Clustering Scheme

**Figure 5:**
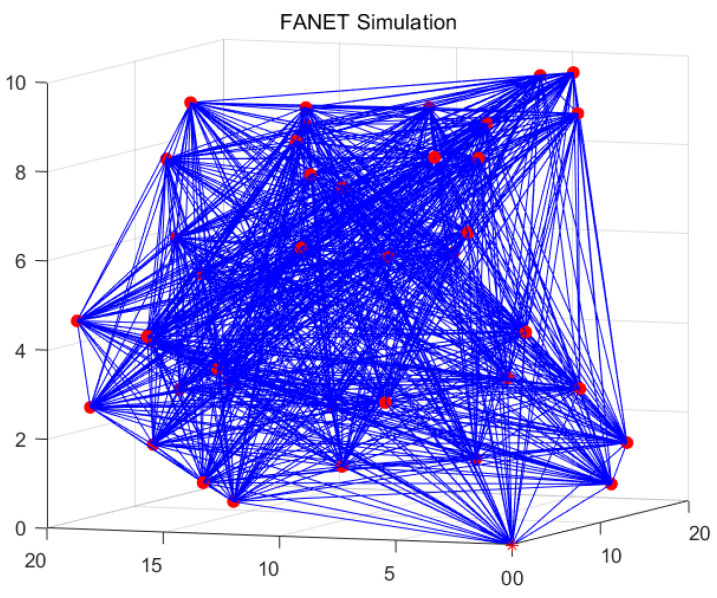
Visualization of UAV network without clustering.

**Figure 6:**
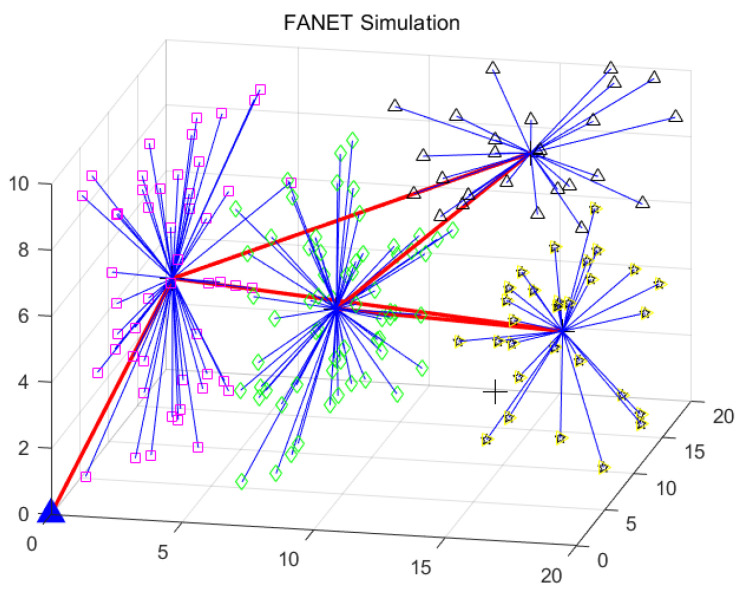
Visualization of the effect of clustering on UAV network.

The FANET working in the ad hoc mode and the clustering-based mode are visualized in Figure 5 and Figure 6, respectively. The UAV network after clustering is connected and orderly, compared with the UAV network before clustering. By the exploitation of the clustering-based architecture, the network overhead and latency can be reduced. The specific analyses are provided in the following experiments.

The performance of IWLC is evaluated under the condition of different weight settings and different numbers of UAVs. The maximum speed of UAVs is 30 m/s, and the transmission range is 300 m. Here, we select four different sets of weight values for comparison in Table 4. The first group of weight settings is determined based on the maximum entropy method [32,33]. For the third group, the four weights are set equally. For the second and fourth groups, the four weights are set with biased values.

**Figure 7:**
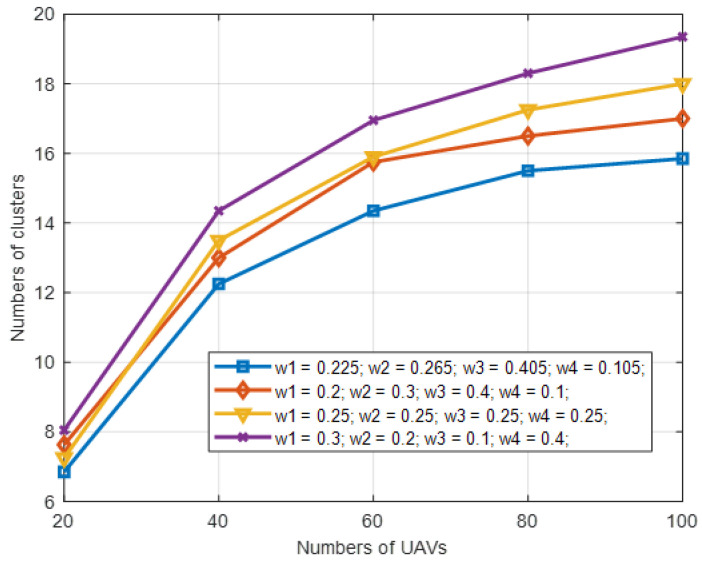
Number of clusters in different weight settings with varying numbers of UAV nodes in IWLC scheme.

**Figure 8:**
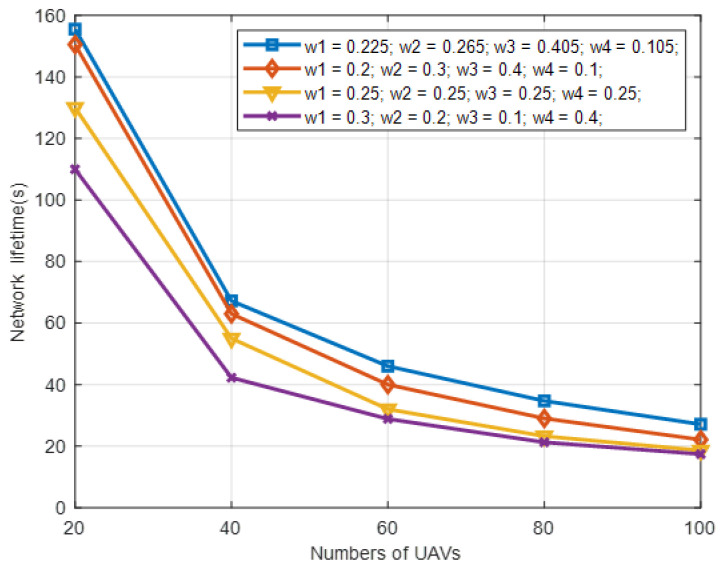
Network lifetime in different weight settings with varying numbers of UAV nodes in IWLC scheme.

As can be observed from Figure 7 and Figure 8, the first group of weight settings outperforms the others on the number of clusters and network lifetime, where the number of clusters refers to the actual number of clusters K, and the network lifetime refers to the occurrence time of the first UAV node running out of energy in the network. The reason for this is that the effect of the four physical factors on the performance is ranked as *M*_*i*_ > *N*_*i*_ > *E*_*i*_ > *D*_*i*_. We set the four weights with the values provided by the first group in the rest of the simulation experiments, which not only matches the ranking, but also optimizes the tradeoff among these physical factors.

### 5.3. Clustering Scheme Comparison

#### 5.3.1. Analysis of Network Density

The performance of IWLC is examined and compared with the clustering scheme of WSCA and ACO, by varying the network density. We evaluate the impact of the network density on the clustering scheme, by varying the number of UAVs. The maximum speed of UAVs is 30 m/s.

**Figure 9:**
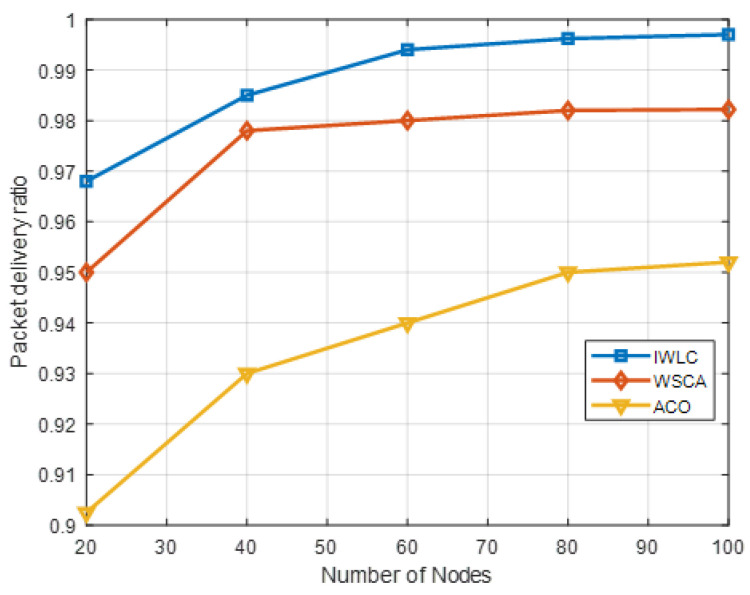
PDR in different clustering schemes with varying numbers of UAV nodes.

Figure 9 compares the PDR of the IWLC with that of the WSCA and ACO. The PDR is defined by

(31) $$PDR=\frac{P_{R}}{P_{S}}$$

where $P_{R}\;$represents the number of packets successfully received by the destination nodes, and $P_{s}\;$represents the total number of packets generated by the source nodes. The destination nodes are the CHs and GCS. The UAV nodes in each cluster send data packets to the corresponding CH, and CHs forward the data packets to the GCS. The higher the PDR, the better the communication quality. The UAV nodes’ distribution is uneven, and the relative mobility is not regular when the number of nodes is small; thus, the PDR is lower. The PDR of three schemes grows with the increasing number of UAV nodes. The IWLC outperforms the other two schemes with a maximum PDR of around 99.7%, when the number of UAVs increases to 100. The higher number of UAV nodes maximize the probability that transmits packets to the destinations. The reason for this is that the IWLC scheme selects stable CHs to forward packets and adopts an efficient clustering algorithm to connect UAV nodes and GCS. Moreover, the IWLC scheme adopts effective cluster maintenance to avoid network congestion and reduce the probability of link disconnection, which improves the connectivity of UAVs and transmission success ratio.

**Figure 10:**
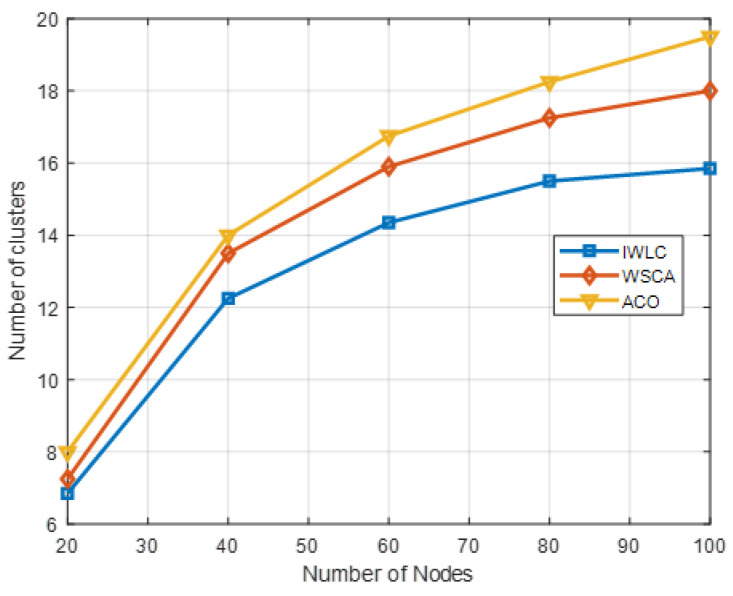
Number of clusters in different clustering schemes with varying numbers of UAV nodes.

Figure 10 shows a comparison of the number of clusters as the number of UAVs varies. The number of clusters for three schemes is constantly growing with the increasing number of UAV nodes in the network. It is clear that the proposed IWLC forms fewer numbers of clusters than the other two schemes. It is because uniform clustering prevents the existence of clusters with isolated nodes or few numbers of nodes, which optimizes the clustering structure. The number of clusters is fewer than the initial optimal value $k^{*}.\;$It could bring an excessive load on the CHs with the increasing number of CMs. However, it can be observed from the IWLC scheme that the growth trend of the number of clusters is more stable, which can better cope with the increasing number of nodes and improve the scalability of the clustering network. In addition, in the cluster maintenance phase, the scheme takes into account the changes and movement of nodes to keep the stability of each cluster in the network.

**Figure 11:**
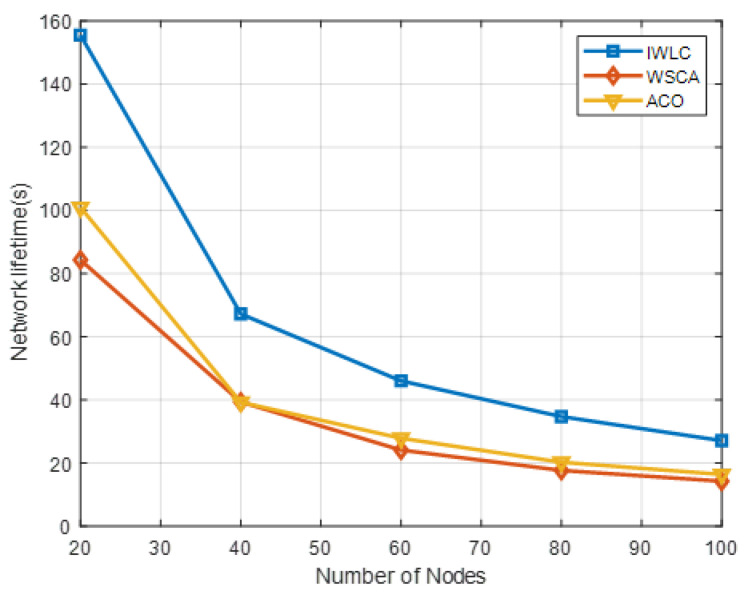
Network lifetime in different clustering schemes with varying numbers of UAV nodes.

Figure 11 shows the performance comparison of IWLC with WSCA and ACO in network lifetime, by varying the number of UAVs. The network lifetime of the clustering schemes decreases, owing to the larger number of UAVs changing the mobility and stability of the network topology with increasing network density. The IWLC has the longest network lifetime compared with the other two schemes, due to its comprehensive CH selection algorithm and cluster maintenance. The proposed scheme fully considers the remaining energy and relative mobility of UAVs, and the weight parameter settings of them are relatively large, which could adapt to the frequently changing energy consumption and network topology. The proposed scheme significantly improves the robustness and adaptability of the network.

**Figure 12:**
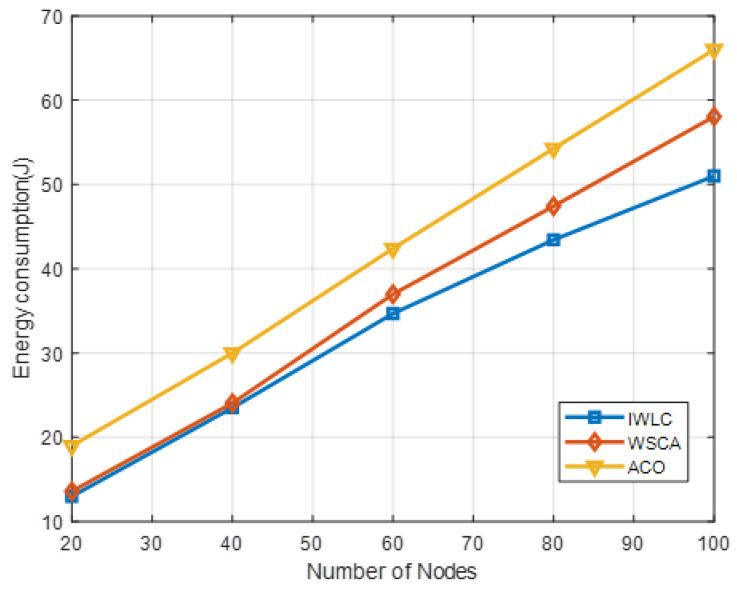
Energy consumption in different clustering schemes under different numbers of UAV nodes.

Figure 12 depicts the energy consumption under different numbers of UAV nodes. It indicates that the energy consumption of the proposed IWLC scheme is the lowest, as compared to the other two algorithms. The energy consumption of IWLC and WSCA is close when the number of UAV nodes varies from 20 to 40. However, as the number of nodes increases, the energy consumption of IWLC is less than that of WSCA, because of its efficient CH selection and cluster maintenance algorithms. The effective packet transmission reduces the extra energy consumption on communications.

#### 5.3.2. Analysis of UAV Speed

**Figure 13:**
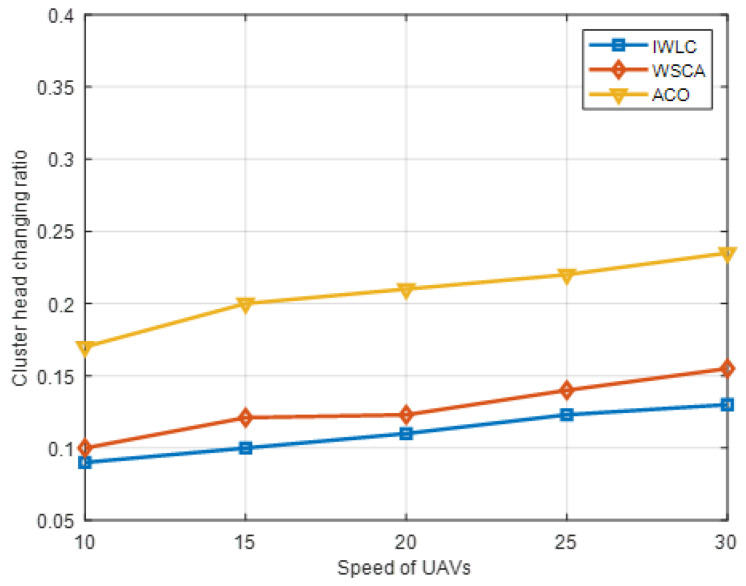
Cluster head changing ratio in different schemes with varying speeds of UAV nodes.

The performance of IWLC is evaluated and compared with the clustering scheme of WSCA and ACO, by varying the speed of the UAVs. The number of UAVs is 100. As can be observed from Figure 13, the cluster head changing ratio grows, owing to the rapidly changing topology and link instability with the increasing speed of the UAVs, and the cluster head changing ratio of the IWLC scheme was always at the minimum. Since the IWLC considers the weight of the four important parameters comprehensively in the CH selection phase, especially the relative mobility and adaptive node degree of the UAVs, the stability of the CHs is better than other schemes. In addition, the scheme adopts a stable clustering formation and cluster maintenance strategy to reduce the replacement frequency of CHs; thus, the selected CHs can better adapt to the fast-speed movement scene.

**Figure 14:**
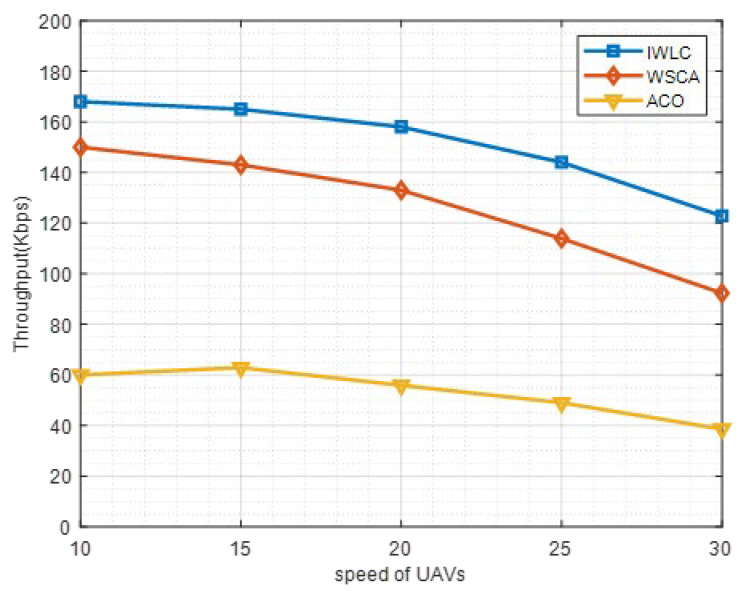
Throughput in different schemes with varying speeds of UAV nodes.

Throughput is an importance metric to assess the performance of the clustering network. In this work, throughput is evaluated by the total amount of data transferred from the source UAV nodes to destinations within a time period. The throughput under different speeds of UAVs is provided in Figure 14. As shown in Figure 14, the throughput of IWLC and WSCA decreases with the speed of UAVs increasing from 10 m/s to 30 m/s, and the throughput of ACO is relatively lower and starts to reduce from 15 m/s to 30 m/s. This is because the frequency of the link disconnection and instability of clustering structure grow, with the increasing speed of the UAV nodes. The proposed IWLC scheme outperforms the other two schemes because the IWLC scheme selects more stable CHs for cluster management and maintenance. Moreover, the effective cluster maintenance strategy of IWLC scheme reduces the probability of communication link disconnection.

#### 5.3.3. Time Complexity Analysis

The time complexity of Algorithm 1 K-means++ Clustering in the proposed IWLC scheme is O(KNI), where N is the number of UAVs, K is the number of clusters, and I is the number of iterations which is set to 50 in the simulation. The time complexity of Algorithm 2 Cluster Head Selection is $O\left(K*\frac{N}{K}\right)=O\left(N\right)$. The time complexity of Algorithm 3 Cluster Maintenance is dominated by Algorithm 2.

Table 5 summarizes the time complexity of the proposed IWLC scheme and the two benchmark methods, namely WSCA and ACO. It can be observed that the proposed IWLC scheme enhances the performance of the number of clusters, PDR, network lifetime, cluster head changing ratio, and energy consumption with equivalent time complexity.

## 6. Conclusions

Owing to the high speed of UAVs and dynamically changing network topology, the traditional clustering schemes cannot be applied to FANETs directly. In this paper, we have proposed the IWLC scheme to enhance the performance of the networks, within the resource limitations. A location-based K-means++ clustering algorithm was firstly developed to form the initial UAV clusters, and a weighted summation-based cluster head selection algorithm was proposed, considering the remaining energy ratio, adaptive node degree, relative mobility, and average distance. The simulation results indicated that, as compared to the WSCA and ACO algorithms, the proposed IWLC scheme enhanced the performance of the number of clusters, packet delivery ratio, network lifetime, cluster head changing ratio, throughput and energy consumption dramatically.

## Figures and Tables

**Table 1:** IWLC performance positioning with other clustering schemes in FANETs.

Proposals	Method	Clustering Overhead	Location Awareness	Stability	Mobility	Energy Efficiency	PDR	CH Chan-ging Ratio	Communicat-ion Safety
LEACH-C [8]	Centralized	High	✓	✓	✓	✗	✗	✓	✗
HEED [9]	Distributed	High	✗	✓	✗	✓	✓	✗	✗
SDN-MQTT [10]	Distributed	Low	✗	✓	✗	✗	✓	✗	✓
CBLADSR [22]	Distributed	High	✓	✗	✓	✓	✓	✗	✗
WSCA [21]	Centralized	Low	✓	✓	✓	✓	✓	✓	✗
EALC [13]	Hybrid	Low	✗	✗	✗	✓	✓	✗	✓
MPCA [14]	Dynamic	High	✓	✓	✓	✗	✗	✓	✗
ACO [23]	Hybrid	Moderate	✗	✓	✓	✗	✓	✓	✗
GWO [24]	Hybrid	High	✗	✗	✓	✗	✓	✗	✗
IWLC	Centralized	Low	✓	✓	✓	✓	✓	✓	✗

**Table 2:** Notations in this paper.

Notation	Meaning	Notation	Meaning
I	UAV ID	$E_{T}$	Total energy consumption
$B_{1}$	Intra-communication bandwidth	$E_{f}$	Flying energy consumption
$B_{2}$	Inter-cluster communication bandwidth	$E_{Tx}$	Transmitting energy consumption
N	Number of UAVs	$E_{Rx}$	Receiving energy consumption
K	Number of clusters	$E_{i}$	Remaining energy ratio of node i
S	Set of UAV nodes	$\varphi_{i}$	Set of neighbor nodes of node i
$k^{*}$	Optimal number of clusters	$Nd_{i}$	Node degree of node i
CH	Cluster Head	$N_{i}$	The adaptive node degree of node i
CM	Cluster Member	$M_{i}$	Relative mobility of node *i*
R	The maximum transmission range of UAVs	$D_{i}$	Average distance of node i and neighbors
C	Set of clustering centers	$v_{i}$	Speed of node i
CL	Set of clusters	$d_{ij}$	Distance between node i and j
$W_{i}$	Weighted summation-based value of node i	GCS	Ground control station
$E_{0}$	Initial energy	PDR	Packet delivery ratio

**Table 3:** Simulation parameters.

Parameter	Value
Network area	2000 m × 2000 m × 1000 m
Simulation time	396 s
UAV transmission range	300 m
Intra-cluster carrier frequency	2.4 GHZ
Inter-cluster carrier frequency	5 GHZ
Communication standard	IEEE 802.11n
Traffic type	CBR
CBR rate	2Mbps
Number of UAVs	20–100
Speed of UAVs	10–30 m/s
Packet size	512 bytes
Initial energy of a node	5 J
Position Update Interval	1 s
Minimum distance between UAVs	5 m
Mobility model	Reference point mobility model
Number of runs	1000
Number of ground stations	1
HELLO message interval	1 s

**Table 4:** Four sets of weight settings.

Group Number	Weight Settings
①	$\omega_{1}=0.225;\omega_{2}=0.265;\omega_{3}=0.405;\omega_{4}=0.105;$
②	$\omega_{1}=0.2;\omega_{2}=0.3;\omega_{3}=0.4;\omega_{4}=0.1;$
③	$\omega_{1}=0.25;\omega_{2}=0.25;\omega_{3}=0.25;\omega_{4}=0.25;$
④	$\omega_{1}=0.3;\omega_{2}=0.2;\omega_{3}=0.1;\omega_{4}=0.4;$

**Table 5:** Time complexity analysis.

Methods	Cluster Formation	CH Selection	Cluster Maintenance	Overall Complexity
IWLC	O(KNI)	O(N)	O(N)	O(KNI)
WSCA	O(KNI)	O(N)	-	O(KNI)
ACO	-	$O\left(N^{2}\right)$	$O\left(N^{2}\right)$	$O\left(N^{2}\right)$

## Data Availability

Data are contained within the article.
